# Pretreatment spatial signature of contralesional cortical activation predicts therapeutic response to 1 Hz rTMS in post-stroke upper limb motor Recovery: A fNIRS-based biomarker study

**DOI:** 10.1016/j.nicl.2025.103917

**Published:** 2025-12-05

**Authors:** Le Jiao, Yuanyuan Tao, Dawei Zhang, Qingmei Chen, Liying Han, Gengrun Tian, Chunlei Shan, Hongjun Zhu

**Affiliations:** a Department of Rehabilitation Medicine, The First Affiliated Hospital of Soochow University, Suzhou 215006, China; b Rehabilitation Center, Tongren Hospital, Shanghai Jiao Tong University School of Medicine, Shanghai 200336, China; c Yuanshen Rehabilitation Institute, Shanghai Jiao Tong University School of Medicine, Shanghai 200025, China

**Keywords:** Repetitive transcranial magnetic stimulation, Functional near-infrared spectroscopy, Stroke rehabilitation, Recovery of Function, Biomarkers

## Abstract

**Background:**

While 1 Hz repetitive transcranial magnetic stimulation (rTMS) targeting contralesional primary motor cortex (M1) shows promise for stroke recovery, individual response variability remains a critical challenge. Emerging evidence suggests that interhemispheric activation patterns may mediate rTMS efficacy. We investigated whether pretreatment spatial features of contralesional activation, measured by functional near-infrared spectroscopy (fNIRS), could predict response to 1 Hz rTMS.

**Methods:**

In this nested unmatched case-control study, 60 patients with upper limb motor impairment received 1 Hz rTMS over contralesional M1 hand area plus conventional rehabilitation for 4 weeks. Responders were defined by a ≥5-point improvement on the Upper Extremity Fugl-Meyer (UEFM) assessment. Cortical activation during affected wrist extension was recorded using fNIRS, and the Euclidean distance from the peak activation channel to the M1 hand area (“activation distance”) was computed. An additional non-rTMS cohort (n=30) receiving only conventional rehabilitation was included to evaluate specificity.

**Results:**

In the rTMS cohort, responders (n=32) exhibited significantly shorter pretreatment activation distances than non-responders (n=28) (25.80±8.82 mm vs. 34.07±7.81 mm; p<0.001). Activation distance independently predicted treatment response after adjusting for baseline UEFM, time since stroke and age (adjusted OR=0.40 per 10 mm increase; 95% CI: 0.17–0.83; p=0.014). A cutoff of ≤25 mm optimally discriminated responders (response rate 86% vs. 34% for >25 mm). No association was found in the non-rTMS cohort, confirming specificity to rTMS response.

**Conclusion:**

Pretreatment contralesional activation proximity to M1—assessed via fNIRS—predicts response to inhibitory rTMS, supporting its use as a biomarker for personalized neuromodulation therapy in stroke rehabilitation.

## 1 Introduction

Stroke is a leading cause of disability worldwide (GBD2019 Stroke Collaborators, 2021), often resulting in hemiparesis or hemiplegia. Repetitive transcranial magnetic stimulation (rTMS) is a promising non-invasive neuromodulation technique used to facilitate motor recovery in stroke patients (Ahmed et al., 2023). By delivering magnetic pulses to the cortex, rTMS modulates cortical excitability and enhances neural plasticity (Jannati et al., 2023). Specifically, 1 Hz rTMS targeting the contralesional primary motor cortex (M1) hand area is a widely used inhibitory protocol to suppress its hyperexcitability. This supposedly alleviates interhemispheric imbalance and promotes the remodeling of ipsilateral corticospinal pathways (Lefaucheur et al., 2020, Xie et al., 2025). ​Despite this strong rationale, the efficacy of 1 Hz rTMS for upper limb recovery is inconsistent (Harvey, R.L., Edwards, D., Dunning, K., Fregni, F., Stein, J., Laine, J., Rogers, L.M., Vox, F., Durand-Sanchez, A., Bockbrader, M., Goldstein, L.B., Francisco, G.E., Kinney, C.L., Liu, C.Y., on behalf of the NICHE Trial Investigators*, Ryan, S., Morales-Quezada, L., Worthen-Chaudhari, L., Labar, D., Schambra, H., Kappy, C.R., Kissela, B., Pratt, W et al., 2018, Kim et al., 2020).

A clue to resolving this inconsistency may come from other clinical domains. In psychiatric applications, individual differences in brain activity patterns have been recognized as key predictors of rTMS efficacy for disorders such as depression and schizophrenia (Dong et al., 2024, Zrenner and Ziemann, 2024). It is therefore reasonable to propose that the variability in motor outcomes post-stroke may similarly arise from individual neurophysiological differences. Identifying the relevant neuroimaging markers to test this possibility is thus critical for personalizing and optimizing rTMS therapy after stroke (Siddiqi et al., 2023).

The interhemispheric competition model posits that contralesional M1 overactivation inhibits ipsilesional neuroplasticity (Di Pino et al., 2014). Based on this model, some studies have adopted 1 Hz rTMS targeting the contralesional M1 hand area in stroke patients to suppress this overactivation and promote ipsilesional neuroplasticity (Chen et al., 2024, Tang et al., 2024). However, functional neuroimaging studies have revealed that contralesional hemisphere overactivation is not limited to the contralesional M1. When stroke patients attempt to move their affected upper limbs, multiple brain regions in the contralesional hemisphere, such as the M1, primary somatosensory cortex, dorsal premotor cortex, and supplementary motor cortex, exhibit increased activation compared to healthy individuals (Chunyong et al., 2023, Dodd et al., 2017). Such overactivation is greater in those with severer motor impairment (Ward et al., 2006), and stimulation of non-M1 contralesional regions, such as the premotor cortex and primary somatosensory cortex, can also produce significant group-level functional improvements (Gao et al., 2024, Lüdemann-Podubecká et al., 2016).

Collectively, these findings imply that contralesional overactivation constitutes a critical substrate for the therapeutic effects of rTMS. A key characteristic of this overactivation is its spatially variable profile across individuals, which is thought to reflect different neuroplastic reorganization strategies (Rehme et al., 2012). We reasoned that the specific location of the peak activation within the contralesional cortex could be a key determinant of rTMS efficacy. We hypothesized that when the peak of contralesional overactivation is spatially proximal to the standard M1 hand area target, the maladaptive process is primarily localized within or near the core motor network. In this scenario, inhibitory rTMS applied to the M1 hand area is likely to directly and effectively suppress the primary source of interhemispheric inhibition (Du et al., 2019). Given that the conventional 1 Hz rTMS protocol for post-stroke upper limb motor dysfunction targets the M1 hand area (Lefaucheur et al., 2020), it remains unclear whether its effectiveness is influenced by these individual differences in pre-treatment brain activation patterns.

Advances in neuroimaging devices have facilitated the capture of pretreatment brain activation data in real clinical settings. Functional near-infrared spectroscopy (fNIRS) is a non-invasive, portable neuroimaging technique that measures cortical activity by detecting concomitant changes in oxygenated (HbO) and deoxygenated (HbR) hemoglobin levels (Chance et al., 1993). This approach of capturing the hemodynamic response to neuronal activity provides a favorable balance between temporal resolution and spatial specificity. While electroencephalography provides superior temporal resolution, fNIRS was chosen for its greater practicality and robustness in our study context, as it is less susceptible to motion artifacts and electromyographic interference from upper limb movements—a critical advantage when testing stroke patients (Pinti et al., 2020). Compared to functional magnetic resonance imaging (fMRI), fNIRS is more accessible, less restrictive, and better suited for task-based studies in clinical populations (Pinti et al., 2020). It has been increasingly used to study motor-related cortical reorganization in stroke, providing valuable insights into both hemispheric activation patterns and their variability across individuals (Kim et al., 2024). Importantly, fNIRS enables the mapping of localized activation changes during the execution of motor tasks that involve the M1 and surrounding regions (Liu et al., 2024), making it a valuable tool to investigate brain activation patterns and guide individualized non-invasive neuromodulation after stroke (Huo et al., 2024).

We hypothesized that the spatial proximity of contralesional peak activation to M1 hand area reflects individual-specific neuroplastic reorganization, with closer proximity enhancing sensitivity to inhibitory rTMS and therapeutic responses. To test this hypothesis, we conducted a nested unmatched case-control study involving patients undergoing 1 Hz rTMS treatment (1 Hz rTMS cohort), focusing on the relationship between fNIRS-derived activation characteristic and therapeutic response to 1 Hz rTMS. To demonstrate that this relationship is specific to 1 Hz rTMS treatment and not a general prognostic marker for conventional rehabilitation, we included an additional cohort of patients who received conventional rehabilitation alone (non-rTMS cohort) for specificity analysis.

## 2 Methods

### 2.1 Study design and participants

Sixty patients with post-stroke upper limb motor impairment, scheduled to receive 1 Hz rTMS targeting the contralesional M1 hand area, were recruited from the Department of Rehabilitation Medicine, First Affiliated Hospital of Soochow University, forming the 1 Hz rTMS cohort. All patients underwent pretreatment task-state fNIRS brain imaging, as well as Upper Extremity Fugl-Meyer (UEFM) assessment at baseline and 4 weeks posttreatment. A nested unmatched case-control design was used to identify brain activation markers associated with response to rTMS treatment. The primary outcome was the change in UEFM score from baseline to 4 weeks posttreatment; patients were classified as responders (≥5-point improvement) or non-responders (<5-point improvement) based on the predefined minimal clinically important difference (MCID) (Page et al., 2012). Exposure factors included fNIRS activation magnitudes across all channels and the distance between the contralesional peak activation site and the M1 hand area (hereafter referred to as activation distance) (Astrakas et al., 2021). An additional 30 patients with post-stroke upper limb motor impairment who did not receive rTMS treatment (non-rTMS cohort) were recruited separately to evaluate the independent association between activation parameters and motor recovery mediated by non-rTMS interventions.

Both cohorts adhered to identical, pre-defined inclusion/exclusion criteria. Inclusion criteria comprised: (1) adults ≥ 18 years with first-ever ischemic stroke/intracerebral hemorrhage confirmed by cranial CT/MRI, demonstrating unilateral supratentorial lesions; (2) moderate-to-severe upper limb motor impairment (UEFM score < 45/66); (3) no prior neuromodulation (rTMS, transcranial direct current stimulation, or deep brain stimulation) therapy exposure. Exclusion criteria included: (1) major systemic comorbidities (New York Heart Association class III–IV heart failure, Child-Pugh class B/C cirrhosis, or estimated glomerular filtration rate < 30 mL/min/1.73 m^2^); (2) active psychiatric disorders (e.g., schizophrenia) or cognitive impairment (Montreal Cognitive Assessment < 18/30); (3) rTMS contraindications (e.g., seizure history, metallic cranial implants); (4) severe aphasia/visuospatial neglect precluding task compliance.

This study was approved by the Ethics Committee of the First Affiliated Hospital of Soochow University and registered with the Chinese Clinical Trial Registry (No. ChiCTR2400084545). All procedures were conducted in accordance with the Declaration of Helsinki, and informed consent was obtained from all participants.

### 2.2 Procedures

#### 2.2.1 General study flow and assessments

After obtaining informed consent, baseline assessments—including task-state fNIRS scan and UEFM evaluation—were conducted before the start of rehabilitation. All assessments at baseline and the 4-week follow-up were performed by a blinded evaluation team, which operated independently from clinical care providers and was not involved in therapeutic decision-making. For participants who withdrew before the 4-week assessment, their final motor function data were recorded immediately prior to withdrawal. To reduce participant burden, neuroimaging (fNIRS) was limited to baseline measurements, while the observational protocol focused solely on longitudinal tracking of motor function.

#### 2.2.2 rTMS coil localization procedure

rTMS was delivered using a figure-8 coil (each loop with a diameter of 8 cm) connected to a magnetic stimulator (model CCY-IA, Wuhan Yiruide Medical Equipment, Wuhan, China) with a maximum output of 6.0 Tesla. The contralesional M1 hand area was localized via a combination of scalp positioning and a systematic single-pulse TMS mapping procedure (Groppa et al., 2012). The specific protocol was as follows:

1) Initial setup: Participants were seated comfortably with the unaffected hand relaxed. Surface electromyography electrodes were placed on the first dorsal interosseous (FDI) muscle of the unaffected limb. The initial coil position was estimated 1 cm anterior to the vertex and 5 cm lateral to the midline of the contralesional hemisphere.

2) Hotspot mapping: Single-pulse TMS was delivered at a fixed suprathreshold intensity (50 % of maximum stimulator output). The coil position was systematically adjusted in 1 cm steps in anterior, posterior, medial, and lateral directions. At each candidate position, five pulses were delivered. The site that elicited the largest mean amplitude of motor evoked potential (MEP) was designated as the preliminary hotspot.

3) Resting motor threshold (RMT) determination: With the coil fixed at the preliminary hotspot, the RMT was determined. The stimulus intensity was gradually decreased until no MEPs > 50 µV were observed in five consecutive trials, then increased in 1 % steps. The RMT was defined as the lowest intensity required to produce MEPs ≥ 50 µV in at least 5 out of 10 consecutive trials (Rossini et al., 2015).

4) Final hotspot confirmation: The mapping procedure was repeated at an intensity of 120 % RMT to confirm the optimal coil position. The site yielding the largest and most consistent MEPs at this intensity was definitively marked as the target for subsequent 1 Hz rTMS.

#### 2.2.3 Treatment regimens

The 1 Hz rTMS cohort received a treatment regimen consisting of 1 Hz rTMS combined with conventional rehabilitation therapies—including therapeutic exercises, occupational therapy, and physical agent modalities—administered once daily, 5 days per week. The stimulation parameters were as follows: frequency of 1 Hz, intensity at 90 % of the individual's RMT, a total of 1200 pulses per session, delivered over 20 min (Liepert et al., 2007). The non-rTMS cohort received conventional rehabilitation therapy alone, following an identical schedule.

### 2.3 fNIRS data acquisition and processing

#### 2.3.1 Task paradigm design

The upper limb motor task was adapted from a previously established paradigm (Hannanu et al., 2020). It consisted of a brief practice/familiarization phase followed by the formal assessment phase.

Practice/familiarization phase: Before the fNIRS recording, all participants first performed the task with their unaffected limb. This step served two key purposes: 1) to ensure that participants fully understood the instructions and the visual cue system, and 2) to familiarize them with the task demands, thereby reducing potential novelty or practice effects during the formal scan. This was particularly important for patients with significant motor impairment, as it allowed us to confirm that any subsequent difficulty in performing the task with the affected limb was primarily due to motor dysfunction rather than a lack of task comprehension.

**Fig. 1:**
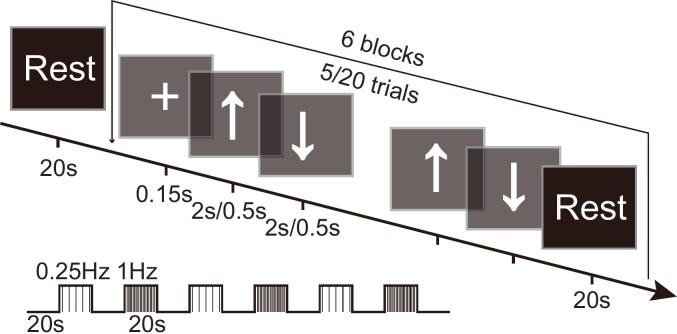
Schematic illustration of the wrist extension task paradigm. Each experimental run consisted of 3 slow-paced blocks and 3 fast-paced blocks with an interblock interval of 20 s. Participants were instructed to extend their wrist upwards when an upward arrow (“↑”) appeared on the screen and flex downwards when a downward arrow (“↓”) was displayed. In the slow-paced blocks, participants performed 5 wrist extension trials at a frequency of 0.25 Hz, while in the fast-paced blocks, they performed 20 trials at 1 Hz.

Formal assessment phase: Immediately after the practice phase, participants underwent the fNIRS recording while performing the same task with their affected limb. The task comprised six blocks of wrist extension: three slow-paced blocks (0.25 Hz) and three fast-paced blocks (1 Hz). Each block lasted 20 s, interspersed with 20-second rest periods. Participants sat in a quiet room with their palms resting on a flat surface. Movements were cued by arrows (“↑” for extension, “↓” for flexion) displayed on a screen. The display duration was 2 s per arrow for the 0.25 Hz blocks and 0.5 s for the 1 Hz blocks. Participants who could not achieve full wrist extension were instructed to attempt the movement to the best of their ability in time with the cues (Lo et al., 2024). The paradigm was programmed using E-Prime 3.0, with event markers synchronized to the fNIRS device via Wi-Fi. A schematic is provided in Fig. 1.

This dual-speed design was implemented to enhance the robustness of cortical activation elicitation across patients with varying degrees of motor impairment, as some may better engage with slower or faster pacing cues. The data from all active blocks (both speeds) were combined for subsequent analysis to derive a comprehensive measure of task-related brain activation.

#### 2.3.2 fNIRS measurement

**Fig. 2:**
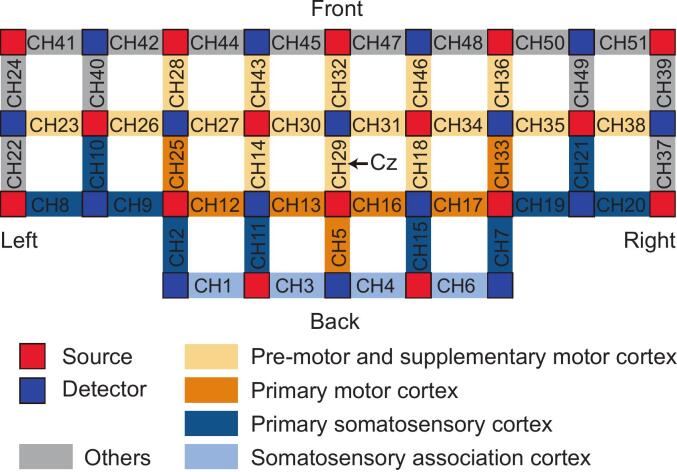
fNIRS channel distribution. The red squares represent light sources, and the blue squares represent detectors. The optical fiber cap was composed of 16 light sources and 16 detectors, forming 51 channels. Each source-detector pair was separated by a distance of 3 cm. Channel 29 was aligned to Cz point, and the optical fiber cap under this arrangement covered 5 regions of interest in each hemisphere. (For interpretation of the references to colour in this figure legend, the reader is referred to the web version of this article.)

A 51-channel fNIRS system (model BS-3000, Wuhan Znion Medical Technology Co., Wuhan, China) was used to measure relative changes in hemoglobin concentrations in the cortical region adjacent to the central sulcus. The sampling rate was set to 20 Hz, with near-infrared light wavelengths of 690 and 830 nm. Each channel, consisting of a source-detector pair with a 3-cm separation, was positioned according to the 10–20 system (with channel CH29 located at the Cz point). A total of 16 light sources and 16 detectors formed these 51 channels. For fNIRS channel normalization, a 3D digitizer (NirMap, Wuhan Union Medical Technology Co., Wuhan, China) recorded the precise spatial coordinates of 4 reference points (Nz, Cz, AL, and RL) and 32 probes (16 light sources and 16 detectors). These 51 channels were then transformed to Montreal Neurological Institute (MNI) space using NIRS-SPM toolbox (Ye et al., 2009). Based on the Brodmann probabilistic atlas, all 51 channels were classified into five regions of interest (ROIs): premotor and supplementary motor cortex (PM/SMC), M1, primary somatosensory cortex (S1), somatosensory association cortex (SAC), and other regions. The corresponding ROI for each channel, along with the arrangement of sources and detectors, is illustrated in Fig. 2.

#### 2.3.3 Signal preprocessing

Since the affected side was not consistent across all participants, fNIRS channels were flipped for individuals with left-sided paresis prior to data preprocessing. For consistency, the right hemisphere was defined as the contralesional, and the left hemisphere as the ipsilesional (Kim et al., 2024). The signal quality of each channel was assessed by calculating the coefficient of variation (CV) of raw signals during the baseline period, with a threshold of 0.25; channels with CVs exceeding this threshold were classified as bad channels. Participants with more than 10 % bad channels were excluded from the analysis. Data preprocessing was conducted using the Homer2 toolbox (Huppert et al., 2009), following this workflow:

1) Optical intensity (raw data) was converted to optical density.

2) Motion artifacts were defined using a sliding window method (window width: 0.5 s, standard deviation threshold: 20.0, amplitude threshold: 5.0).

3) Motion artifacts were corrected via cubic spline interpolation.

4) A bandpass filter (0.01–0.1 Hz) was applied to reduce signal noise.

5) Finally, the optical density was converted to concentrations of HbO and HbR based on the modified Beer-Lambert law, with the partial pathlength factor set to 6.

#### 2.3.4 First and second-level analysis of task-state fNIRS

As previous studies have shown, changes in HbO concentration more sensitively reflect cortical activity; thus we used HbO as an indicator to assess cortex activation (Cui et al., 2010). The NIRS-KIT toolbox was utilized for both first-level (individual) and second-level (group) analysis (Hou et al., 2021).

At the first level, a general linear model (GLM) was employed to analyze task-related activation (Tak and Ye, 2014). The analysis used the entire, continuous preprocessed HbO time series for each channel. The model was constructed based on the block design described in Section 2.3.1. A single task regressor was created by convolving a boxcar function (with a duration of 20 s, aligned to the onset of each of the six active blocks) with the canonical double-gamma hemodynamic response function. The rest periods served as the implicit baseline. The model is expressed as:

Y=Xβ+ε(Equation1)

where Y represents the preprocessed HbO time series of each channel; X denotes the task regressor (as defined above), along with a constant term to model the baseline signal; β refers to the coefficient corresponding to the task regressor, indicating the magnitude of task-evoked HbO concentration changes; and ε represents the residual time series. The β values were estimated using the Ordinary Least Squares method.

The residual time series, defined as the difference between the observed values and the model-predicted values (*Equation 2*), reflects task-unrelated spontaneous neural activity (Zhao et al., 2023). Interhemispheric functional connectivity was assessed by calculating the Pearson correlation coefficient between the residual time series of symmetrical channels across hemispheres.

$$\varepsilon =Y-X\hat{\beta }\left(Equation2\right)$$

At the second level, assuming that spontaneous HbO fluctuations were unrelated to the task (β = 0), one-sample t-tests were conducted to determine whether β values within group differed significantly from zero. Independent samples t-tests were used to compare activation magnitudes across channels between groups. Multiple comparison corrections were performed using the false discovery rate (FDR) method to determine the activation status of each channel at the group level.

#### 2.3.5 Contralesional hemisphere activations

Pretreatment differences in the magnitude and location of cortical activation were compared between patients with varying treatment responses during follow-up. For each fNIRS channel, activation magnitude was quantified using the β values derived from the GLM analysis. The spatial overlap between activation and the rTMS stimulation target (M1 hand area) was quantified by *activation distance*—defined as the Euclidean distance between the MNI coordinates of the channel with the peak β value and the standard MNI coordinates [40, −20, 52], corresponding to the M1 hand knob (Yousry, 1997).

### 2.4 Statistical analysis

An a priori sample size was calculated using PASS 2021 software. Using the upper bound (0.68) of the 95 % confidence interval (0.43–0.68) from a previous study at our center (Zhu et al., 2025) as the response probability (P_1_) for the 1 Hz rTMS cohort, and assuming an odds ratio (OR) of 0.4 per standard deviation increase in activation distance (based on unpublished preliminary data), a sample size of 50 participants was required to achieve 80 % power at a two-sided alpha of 0.05. After accounting for an anticipated dropout rate of 15–20 %, the final target sample size was set at 60.

The association between activation distance (rescaled to 10-mm units) and treatment response was assessed using Firth penalized-likelihood logistic regression to reduce small-sample bias (Firth, 1993). Covariates for adjustment were selected a priori based on previous literature and clinical rationale. Only covariates with well-established significant impacts on post-stroke motor recovery were included in the multivariate model, while those with controversial evidence were omitted. The model was adjusted for pre-specified covariates—baseline UEFM score, time since stroke, and age (Huang et al., 2009, Shin et al., 2022, Sikuka et al., 2024)—yielding an adjusted OR for activation distance that was independent of these confounders.

To assess the independent association of activation distance with motor recovery and evaluate its moderating effect on 1 Hz rTMS responses, data from the non-rTMS cohort were analyzed separately. Subsequently, data from both cohorts were pooled, and an interaction term between rTMS treatment and activation distance was included in the weighted regression model. This model was adjusted for the pre-specified covariates to ensure that the estimated interaction effect was robust to potential confounding by these variables.

Receiver operating characteristic (ROC) curve was utilized to determine the optimal cutoff value of activation distance for predicting rTMS treatment response, based on the maximal Youden Index. Missing values were imputed using the last observation carried forward method. We conducted sensitivity analyses to evaluate the robustness of our findings, including (1) multiple imputation by chained equations (MICE) and (2) complete-case analysis (see details in supplement 1). A two-sided p-value < 0.05 was considered statistically significant. All analyses were performed using R software (version 4.3.1).

## 3 Results

### 3.1 Patient and treatment characteristics

Of the 60 participants in the 1 Hz rTMS cohort, four withdrew before the 4-week UEFM assessment. For these patients, their last available UEFM scores were imputed as week 4 scores, given that they exhibited significant improvement (UEFM change ≥ 5 points) prior to withdrawal. All 60 participants completed baseline fNIRS assessments with valid data (the number of bad channels did not exceed the 10 % threshold; maximum: 4 channels, 8 %).

Following 1 Hz rTMS treatment, 28 participants were classified as non-responders and 32 as responders. Baseline characteristics of these two groups are presented in Table 1. Responders had significantly lower baseline UEFM scores, and a higher proportion of recent stroke (within 6 months) compared to non-responders (p < 0.05). After 4 weeks of treatment, the UEFM score of responders was 37.16 ± 19.79, while that of non-responders was 15.41 ± 17.29.

The separately recruited non-rTMS cohort (n = 30) and the 1 Hz rTMS cohort demonstrated comparable baseline characteristics, as confirmed by statistical comparison presented in Table S1 (supplement 1).

### 3.2 Comparison of fNIRS activation parameters between responders and non-responders in the 1 Hz rTMS cohort

**Fig. 3:**
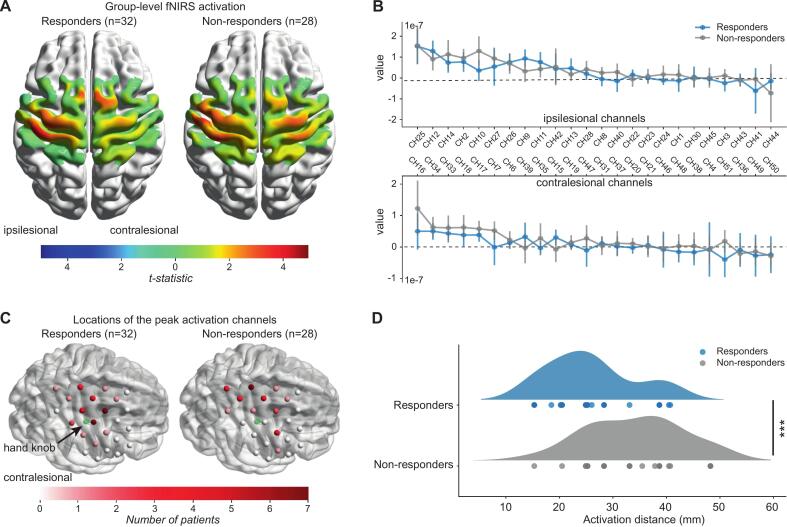
Comparison of fNIRS cortical activation patterns between responders and non-responders in the 1 Hz rTMS cohort. (A) One-sample *t*-test maps of β values (vs. zero) for responders and non-responders. Significant task-evoked activations (FDR-corrected p < 0.05) were observed in bilateral hemispheres for both groups. (B) Group comparison of β values across channels. No significant differences in activation magnitude were observed between responders (blue bars) and non-responders (grey bars) after FDR correction (all p > 0.05). Error bars represent 95 % confidence intervals. (C) The frequency map of individual peak activation coordinates in the contralesional hemisphere. The intensity of red indicates the number of patients with peak activation in that channel. The green point, marked by an arrow, denotes the M1 hand area. Responders showing a higher concentration near the M1 hand area. (D) Activation distance (Euclidean distance between peak activation site and M1 hand area) was significantly shorter in responders vs. non-responders. ***p < 0.001. (For interpretation of the references to colour in this figure legend, the reader is referred to the web version of this article.)

Fig. 3A illustrates task-evoked group-level activation t-maps for responders and non-responders. Both groups exhibited significant task-evoked activations (FDR-corrected p < 0.05) in the bilateral M1 and PM/SMC (channel details in Table S2, supplement 1). However, direct comparisons of activation magnitude for each channel revealed no statistically significant differences between the two groups (Fig. 3B).

The cortical distribution of peak activation sites differed between responders and non-responders (Fig. 3C). Responders demonstrated a higher concentration of peak activation near the M1 hand area, whereas non-responders exhibited more dispersed activations further from this region. Notably, the mean activation distance was significantly shorter in responders (25.80 ± 8.82 mm) than in non-responders (34.07 ± 7.81 mm; t = -3.85, p < 0.001) (Fig. 3D). Details of peak activation channels and their corresponding frequencies are provided in Table S3 (supplement 1).

Post hoc analysis comparing cortical activation between the slow (0.25 Hz) and fast (1 Hz) movement conditions revealed no statistically significant differences in activation magnitude or the location of peak activation (all p > 0.05, see Supplementary Fig. S1), indicating that the spatial signature of contralesional activation was consistent across task parameters.

### 3.3 The predictive and moderating role of activation distance in rTMS response

We first assessed whether activation distance was associated with treatment response within the 1 Hz rTMS cohort. Univariate logistic regression revealed that baseline UEFM score, time since stroke, and activation distance were significantly associated with treatment response. Multivariate logistic regression, adjusted for baseline UEFM score, time since stroke and age, confirmed that activation distance was independently associated with treatment response. Specifically, for every 10 mm increase in activation distance, the odds of responding to rTMS decreased by 60 % (adjusted OR = 0.40, 95 % CI: 0.17–0.83, p = 0.014) (Table 2).

**Fig. 4:**
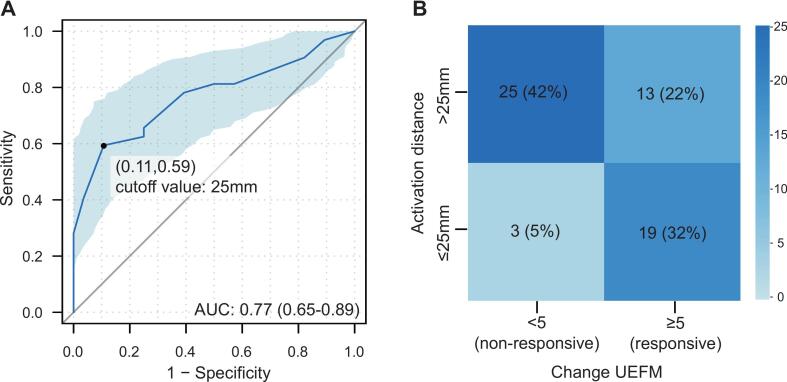
Predictive performance of activation distance for rTMS response. A) ROC analysis identified an optimal activation distance cutoff of 25 mm. B) Confusion matrix showing classification accuracy using the 25 mm cutoff.

ROC analysis identified the optimal cutoff value of activation distance for predicting rTMS treatment response as approximately 25 mm (Fig. 4A). Among patients with an activation distance ≤ 25 mm, the rTMS response rate was 86 % (95 % CI: 65 %–97 %); in contrast, the response rate was only 34 % (95 % CI: 20 %–51 %) for those with a distance > 25 mm (confusion matrix in Fig. 4B).

**Fig. 5:**
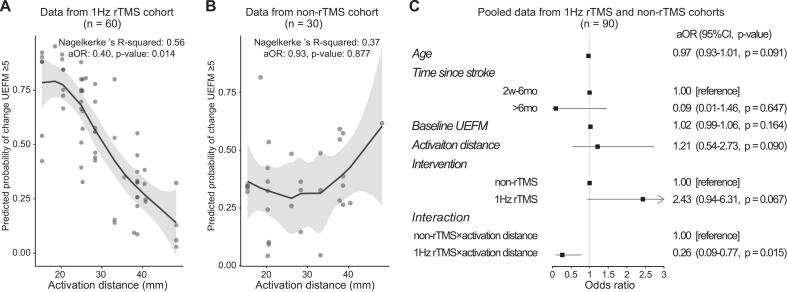
Moderating effect of activation distance on rTMS efficacy. (A) In the 1 Hz rTMS cohort, shorter activation distance predicted higher odds of successful response. (B) No association was observed in the non-rTMS cohort. (C) Interaction analysis (pooled cohorts) confirmed a significant rTMS × activation distance effect (p < 0.05), indicating activation distance moderates rTMS efficacy. Error bars: 95 % CI. All analyses, including those for individual cohorts and the pooled dataset, were adjusted for baseline Upper Extremity Fugl-Meyer score, time since stroke, and age.

To further investigate the specificity of this association to rTMS treatment, we compared the findings with a non-rTMS cohort. In the 1 Hz rTMS cohort, a shorter activation distance was significantly associated with a higher likelihood of treatment response (Fig. 5A). In contrast, no significant association was observed between activation distance and motor improvement (UEFM change ≥ 5) in the non-rTMS cohort (aOR = 0.93, 95 % CI: 0.34–2.31, p = 0.877) (Fig. 5B).

Analysis of the pooled dataset revealed a significant interaction between rTMS treatment and activation distance after adjusting for baseline UEFM score, time since stroke, and age (Fig. 5C), indicating that activation distance moderates the effect of rTMS on motor recovery. Without rTMS treatment, the activation distance alone was not a significant predictor of motor improvement.

### 3.4 Relationship between peak activation variability and interhemispheric functional connectivity

**Fig. 6:**
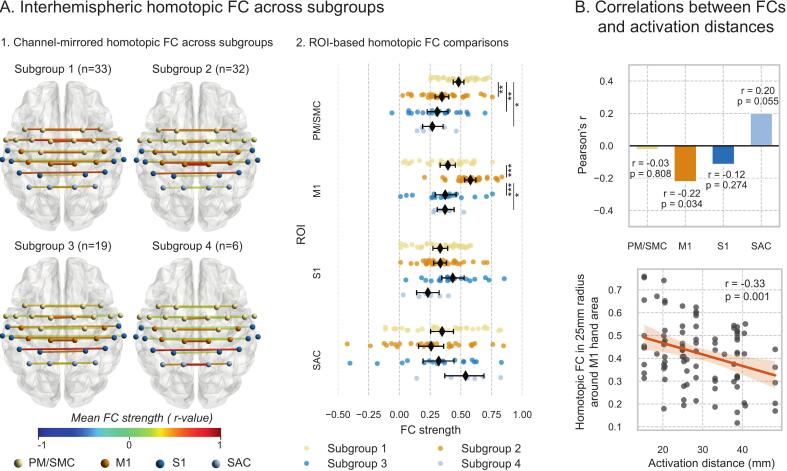
Interhemispheric FC in homologous ROIs across peak-activation subgroups. Patients were categorized into four subgroups based on the ROI exhibiting peak activation: Subgroup 1 (PM/SMC), Subgroup 2 (M1), Subgroup 3 (S1), and Subgroup 4 (SAC). (A)Visualization of interhemispheric FC across subgroups and inter-subgroup statistical comparisons at the ROI level. Comparisons were performed using one-way ANOVA followed by Tukey’s HSD post-hoc tests. (B)Pearson correlation analysis between interhemispheric FC in each homologous ROI and activation distance. *p < 0.05, **p < 0.01, ***p < 0.001.

In the pooled dataset (n = 90), patients were categorized into four subgroups based on the ROI where peak activation occurred: Subgroup1 (n = 33) with peak activation in the PM/SMC, Subgroup2 (n = 32) in M1, Subgroup3 (n = 19) in S1, and Subgroup4 (n = 6) in the SAC. Significant differences in interhemispheric FC between homologous regions were observed across subgroups. Specifically, interhemispheric FC in the PM/SMC was significantly stronger in Subgroup1 compared to the other subgroups (p < 0.05). Similarly, M1 interhemispheric FC was significantly stronger in Subgroup2 than in the other subgroups (p < 0.05). Although interhemispheric FC in S1 was slightly higher in Subgroup3, the difference did not reach statistical significance compared to the other subgroups. Likewise, interhemispheric FC in the SAC was marginally elevated in Subgroup4 but not statistically significant relative to the other subgroups (Fig. 6A).

A significant negative correlation was observed between M1 interhemispheric FC and activation distance (r = -0.22, p = 0.034). When a spherical ROI with a radius of 25 mm was defined—centered on the hand knob—interhemispheric FC between homologous regions within this ROI demonstrated a stronger negative correlation with activation distance (r = –0.33, p = 0.001) (Fig. 6B).

### 3.5 Sensitivity analyses

Sensitivity analyses, using both MICE and complete-case approaches, affirmed the robustness of the association between activation distance and treatment response. The results were consistent with the primary analysis: the adjusted odds ratio for a 10-mm increase in distance was 0.43 (95 % CI: 0.20–0.90; p = 0.027) with MICE and 0.44 (95 % CI: 0.18–0.88; p = 0.020) with complete-case analysis (Table S4). This demonstrates that the finding is not sensitive to the method of handling missing data.

## 4 Discussion

This study identified a critical spatial signature in contralesional cortical activation that predicts response to 1 Hz rTMS in stroke patients with upper limb motor impairment: shorter Euclidean distances between the peak activation site and the contralesional M1 hand area were associated with greater motor recovery following contralesional M1-targeted 1 Hz rTMS. Notably, this spatial relationship was absent in the non-rTMS cohort, confirming its specificity to rTMS intervention rather than spontaneous recovery or conventional rehabilitation effects.

### 4.1 Contralesional activation patterns and rTMS response: Beyond activation magnitude

Group-level fNIRS analysis revealed robust bilateral sensorimotor activation in both responders and non-responders during affected wrist extension tasks, consistent with the well-documented post-stroke phenomenon of contralesional overactivation (Chollet et al., 1991). This pattern reflects adaptive (or maladaptive) reorganization of bilateral motor networks, yet its functional implications remain debated. The interhemispheric competition model posits that contralesional activation impairs motor function by inhibiting ipsilesional plasticity(Grefkes et al., 2010, Mirdamadi et al., 2023). However, an alternative viewpoint suggests that activation in the contralesional hemisphere may serve a compensatory role for functions associated with the damaged hemisphere (Bütefisch et al., 2005, Schaechter and Perdue, 2008).

Our findings challenge the notion that activation magnitude alone drives rTMS response: no significant differences in activation strength were observed between responders and non-responders across any brain region. This contrasts with smaller studies linking activation magnitude to motor outcomes(Calautti et al., 2007, Ward et al., 2003), but aligns with the hypothesis that rTMS modulates recovery trajectories in ways that decouple baseline activation strength from therapeutic response. Specifically, rTMS may rescue patients with poor prognosis under conventional therapy, obscuring simple correlations between activation magnitude and recovery. This suggests that neuroimaging biomarkers for spontaneous recovery may not generalize to rTMS response prediction—underscoring the need for novel, intervention-specific markers like the activation distance identified here.

### 4.2 Spatial proximity to M1: A mechanistic basis for rTMS efficacy

The key insight of this study is the association between spatial distribution of contralesional activation and rTMS response. Responders exhibited peak activation sites clustered near the contralesional M1 hand knob, whereas non-responders showed dispersed activation further from this region. Quantitatively, a 10-mm increase in activation distance reduced the odds of rTMS response by 60 % (aOR = 0.40), independent of baseline motor function and stroke chronicity.

This spatial relationship likely reflects the mechanistic basis of 1 Hz rTMS: inhibitory modulation of the contralesional M1 to alleviate interhemispheric imbalance(Lefaucheur et al., 2020, Xie et al., 2025). When peak activation is proximal to the M1 hand area, neurons in this region may exert stronger inhibitory control over ipsilesional pathways (Bice et al., 2022), making them more sensitive to rTMS-induced suppression. Conversely, in patients with distal activation (>25 mm), the contralesional M1 may play a minimal role in motor network reorganization—explaining why fixed-site rTMS fails to enhance recovery. This aligns with recent work showing that the functional role of contralesional regions depends on their integration with ipsilesional structures (Mustin et al., 2024), highlighting spatial specificity as a critical determinant of neuromodulation efficacy. Our subsequent analysis of interhemispheric functional connectivity provides further support for this hypothesis, directly linking the spatial distribution of activation to network-level communication between hemispheres.

### 4.3 Interhemispheric functional connectivity: A potential mechanistic link

Our study further revealed a novel neurophysiological correlate of activation distance: interhemispheric FC. Specifically, a shorter activation distance was associated with stronger interhemispheric FC between homologous regions around the contralesional M1 hand area. Previous studies have indicated that homologous interhemispheric FC reflects the integrity of transcallosal interhemispheric fiber pathways (Yuan et al., 2020), which are often disrupted after stroke. Generally, interhemispheric FC (e.g., between bilateral M1 regions) is negatively correlated with stroke severity (Carter et al., 2010). It has been proposed that in patients with relatively mild impairment and preserved structural connectivity, contralesional M1 may exert inhibitory influences on the ipsilesional hemisphere (Suputtitada et al., 2025). Thus, the observed stronger M1–M1 FC associated with shorter activation distance may reflect a stronger inhibitory drive from the contralesional M1, which could be more effectively modulated by inhibitory 1 Hz rTMS—thereby explaining the higher response rate in these patients.

Conversely, a longer activation distance was correlated with weaker M1-M1 FC. This could represent a different, perhaps more compensatory, reorganization strategy where the influence of the contralesional M1 area is less directly inhibitory. Inhibiting a region that is already less functionally connected (and thus less influential) would explain the diminished therapeutic effect of M1-targeted rTMS in these patients.

The subgroup analysis further reinforces the concept of regional specificity. The strongest FC was observed in the homologous regions where the peak activation occurred (e.g., strongest PM/SMC FC in the PM/SMC peak subgroup). This aligns with the idea that the functional role of a contralesional region is tied to its specific connections with the ipsilesional hemisphere (Mustin et al., 2024). Future studies investigating connectivity-based neuromodulation targets may benefit from considering both the spatial location of peak activity and its functional connectivity profile (Aziz et al., 2025).

### 4.4 Clinical implications: Optimizing patient selection for M1-targeted rTMS

The 25-mm activation distance cutoff identified via ROC analysis offers a practical tool to optimize patient selection for contralesional M1-targeted 1 Hz rTMS. This represents a crucial step away from the “one-size-fits-all” paradigm toward a stratified approach, where patients likely to benefit from the standard protocol are identified upfront, thereby maximizing therapeutic efficacy and resource allocation.

For patients with an activation distance > 25 mm, for whom the conventional M1-targeted protocol shows limited efficacy, our findings highlight the limitations of the current standard approach and underscore the need for alternative strategies. While our study was not designed to evaluate alternative targets, the observed dispersion of peak activation in non-responders suggests that their motor recovery might be subserved by contralesional regions beyond M1. This rationale motivates future research to explore whether expanding stimulation targets to non-M1 regions (e.g., the premotor or primary somatosensory cortex) could benefit this patient subgroup (Gao et al., 2024; Lüdemann-Podubecká et al., 2016). Alternatively, a more personalized approach could involve using task-state fNIRS to guide stimulation precisely to the individual’s peak activation site, whether it falls within or outside the standard M1 hand area. Ultimately, our biomarker not only enables better patient stratification for existing protocols but also provides a framework for developing personalized neuromodulation strategies in the future.

### 4.5 Limitations and future directions

Several limitations should be noted. First, the unmatched case-control design, despite adjusting confounders via regression, may not fully eliminate residual bias. Second, the sample primarily included subacute stroke patients, limiting generalizability to chronic populations. Third, we lacked precise coordinates of actual rTMS stimulation sites, which could be addressed via 3D navigated TMS (Tam et al., 2024). Finally, the focus on 1 Hz rTMS means findings may not extend to other frequencies or stimulation paradigms.

Future studies should validate the activation distance biomarker in randomized controlled trials, explore its utility for guiding individualized stimulation targets, and investigate whether combining fNIRS with other modalities (e.g., structural MRI) improves predictive accuracy. Additionally, longitudinal fNIRS monitoring could clarify how rTMS modulates activation topography over time, shedding light on dynamic recovery mechanisms.

In summary, pretreatment fNIRS-derived activation distance emerges as a specific, feasible biomarker for 1 Hz rTMS response. By linking spatial characteristics of contralesional activation to neuromodulation efficacy, this work advances toward personalized stroke rehabilitation—where neuroimaging guides not just whether to use rTMS, but how to target it.

## 5 Conclusion

This study demonstrates that the pretreatment spatial signature of contralesional cortical activation—quantified as the Euclidean distance between the peak activation site and the contralesional M1 hand area (activation distance)—serves as a specific and independent predictor of therapeutic response to 1 Hz rTMS in post-stroke upper limb motor recovery.

Specifically, patients with a shorter activation distance (≤25 mm) showed a significantly higher response rate to contralesional M1-targeted 1 Hz rTMS (86 % vs. 34 % for those with > 25 mm), even after adjusting for baseline motor function, time since stroke and age. Critically, this association was absent in the non-rTMS cohort, confirming that activation distance specifically modulates rTMS efficacy rather than reflecting spontaneous recovery or responsiveness to conventional rehabilitation. Furthermore, the correlation between shorter activation distance and stronger interhemispheric functional connectivity suggests a potential neurophysiological substrate for this spatial biomarker.

These findings address a key clinical challenge in stroke rehabilitation: the high variability in rTMS response. By establishing fNIRS-derived activation distance as a feasible biomarker, our work provides a practical tool to optimize patient selection for 1 Hz rTMS—identifying those most likely to benefit from conventional M1-targeted protocols. For patients with distal peak activation (>25 mm), this biomarker confirms the limited utility of the standard M1-targeted approach and points to an urgent need for future research to individualize rTMS targets (e.g., guided by task-state fNIRS) or explore alternative non-M1 stimulation regions.

Future research should validate this biomarker in large-scale randomized controlled trials, investigate its utility across acute, subacute, and chronic stroke phases, and explore its integration with other neuroimaging modalities (e.g., fMRI) to refine predictive accuracy. Ultimately, such advances may transform stroke rehabilitation from a “one-size-fits-all” approach to personalized neuromodulation, maximizing therapeutic efficacy and improving patient outcomes.

## Ethics statement

This study was approved by the Ethics Committee of the First Affiliated Hospital of Soochow University (Approval No. 2024–056) and registered with the Chinese Clinical Trial Registry (No. ChiCTR2400084545). All procedures were conducted in accordance with the Declaration of Helsinki, and informed consent was obtained from all participants.

## CRediT authorship contribution statement

**Le Jiao:** Writing – original draft, Project administration. **Yuanyuan Tao:** Project administration, Data curation. **Dawei Zhang:** Formal analysis. **Qingmei Chen:** Methodology. **Liying Han:** Software. **Gengrun Tian:** Visualization. **Chunlei Shan:** Writing – review & editing, Funding acquisition. **Hongjun Zhu:** Writing – review & editing, Supervision, Conceptualization.

## Funding

This work was supported by National Natural Science Foundation of China (Grant No. 82272612).

## Declaration of competing interest

The authors declare that they have no known competing financial interests or personal relationships that could have appeared to influence the work reported in this paper

## Data Availability

Data will be made available on request.
