# Supplementary material for: Pretreatment spatial signature of contralesional cortical activation predicts therapeutic response to 1 Hz rTMS in post-stroke upper limb motor Recovery: A fNIRS-based biomarker study
Source: Neuroimage Clin. 2025 Dec 5;49:103917. doi: 10.1016/j.nicl.2025.103917 (PMC12753248; doi:10.1016/j.nicl.2025.103917)
Supplement: Supplementary Data 1 [file mmc1.docx]

Supplementary Information for “Pretreatment Spatial Signature of Contralesional Cortical Activation Predicts Therapeutic Response to 1Hz rTMS in Post-stroke Upper Limb Motor Recovery: A fNIRS-Based Biomarker Study”

***S1. Supplementary Methods***

*S1.1 Detailed description of sensitivity analyses for missing data*

While the Last Observation Carried Forward method was used in the primary analysis for the four participants who withdrew before the 4-week assessment, we recognized the potential limitations of this approach (Little et al., 2012). To rigorously evaluate the robustness of our findings, we conducted comprehensive sensitivity analyses using two alternative approaches: (1) Multiple Imputation by Chained Equations (MICE), and (2) complete-case analysis.

*S1.1.1 Multiple Imputation by Chained Equations (MICE)*

Multiple imputation was performed using the “mice” package in R (Buuren and Groothuis-Oudshoorn, 2011). The imputation model included all variables used in the primary analysis (age, sex, stroke type, lesion side, time since stroke, baseline UEFM score, activation distance) to predict the missing Week-4 UEFM change scores. We generated 10 imputed datasets. Predictive mean matching was used for the continuous UEFM change score to preserve its data distribution.

Within each imputed dataset, treatment response was defined as a binary outcome (UEFM improvement ≥5 points). The association between activation distance and treatment response was assessed using Firth’s penalized-likelihood logistic regression (Heinze, 2006), adjusting for the pre-specified covariates (age, time since stroke, and baseline UEFM score). Results from the 10 datasets were pooled using Rubin's rules to obtain the final odds ratio and confidence interval.

*S1.1.2* *Complete-Case Analysis*

As a complementary approach, we performed a complete-case analysis by excluding the four participants with missing week-4 UEFM scores. The same Firth penalized-likelihood logistic regression model (adjusting for age, chronicity, stroke type, and baseline UEFM score) was applied to the remaining 56 participants to evaluate the association between activation distance and treatment response.

***S2. Supplementary Results***

*S2.1. Comparison of baseline characteristics between the independent cohorts*

An independent cohort of 30 patients receiving only conventional rehabilitation (the non-rTMS cohort) was recruited to evaluate the specificity of the identified biomarker for predicting response to rTMS, rather than general motor recovery. This cohort was frequency-matched to the 1Hz rTMS cohort on key demographic and clinical variables, including sex, lesion side, time since stroke, and lesion type, to enhance group comparability. As presented in Table S1, the two cohorts demonstrated comparable baseline characteristics across all measured variables, including age and baseline upper limb motor impairment (UEFM scores). This supports the use of the non-rTMS cohort for testing the specificity of the association between activation distance and treatment response to the rTMS intervention.

Although the response rate was numerically higher in the 1Hz rTMS cohort (53%) than in the non-rTMS cohort (33%), this difference was not statistically significant (p=0.117, Table S1). This observation underscores the substantial inter-individual variability in response to the 1Hz rTMS protocol, a key challenge that the present study aims to address.

**Table S1.** Baseline and outcome profiles of the study cohorts

| Characteristics | Overall  (n = 90) | non-rTMS cohort  (n = 30) | 1Hz rTMS cohort  (n = 60) | p |
| --- | --- | --- | --- | --- |
| Age, mean (SD), y | 63.46 (14.57) | 65.70 (12.81) | 62.33 (15.36) | 0.304 |
| Sex, n (%) |  |  |  | 1.000 |
| Male | 63 (70%) | 21 (70%) | 42 (70%) |  |
| Female | 27 (30%) | 9 (30%) | 18 (30%) |  |
| Lesion side, n (%) |  |  |  | 1.000 |
| Left | 42 (47%) | 14 (47%) | 28 (47%) |  |
| Right | 48 (53%) | 16 (53%) | 32 (53%) |  |
| Times from stroke, n (%) |  |  |  | 1.000 |
| 2w-6mo | 75 (83%) | 25 (83%) | 50 (83%) |  |
| >6mo | 15 (17%) | 5 (17%) | 10 (17%) |  |
| Lesion type, n (%) |  |  |  | 1.000 |
| Ischemic stroke | 57 (63%) | 19 (63%) | 38 (63%) |  |
| Intracerebral haemorrhage | 33 (37%) | 11 (37%) | 22 (37%) |  |
| Upper extremity Fugl-Meyer, mean (SD) |  |  |  |  |
| Baseline | 21.67 (18.05) | 18.80 (17.50) | 23.10 (18.30) | 0.289 |
| 4 w | 26.04 (19.85) | 22.37 (18.59) | 27.88 (20.36) | 0.216 |
| 4w change baseline | 4.38 (3.24) | 3.57 (2.97) | 4.78 (3.32) | 0.093 |
| Response rate, n (%) | 42 (47%) | 10 (33%) | 32 (53%) | 0.117 |

*S2.2. Group-level activation channels*

Group-level cortical activation during the affected wrist extension task was analyzed separately for responders and non-responders in the rTMS cohort. A one-sample t-test was performed on the beta (β) values from the general linear model for each channel to determine significant task-evoked activation compared to baseline (zero). The false discovery rate (FDR) method was applied to correct for multiple comparisons across the 51 fNIRS channels. Table S3 lists the fNIRS channels that showed statistically significant activation (FDR-corrected p < 0.05) at the group level for responders and non-responders.

**Table S2.** Group-level significantly activated channels in responders and non-responders

| Brain region | Channel | MNI coordinates, mm | | | Responders  (n=32) | | Non-responders  (n=28) | |
| --- | --- | --- | --- | --- | --- | --- | --- | --- |
|  |  | x | y | z | t-statistic | adjusted p | t-statistic | adjusted p |
| Ipsilesional |  |  |  |  |  |  |  |  |
| Pre-motor and supplementary motor cortex | CH26 | -52 | 1 | 53 | 3.16 | 0.018 | 3.43 | 0.020 |
|  | CH14 | -24 | -13 | 75 | - | - | 3.88 | 0.010 |
|  | CH27 | -34 | 1 | 66 | 3.96 | 0.006 | - | - |
| Primary motor cortex | CH25 | -46 | -12 | 62 | 3.75 | 0.007 | 3.27 | 0.021 |
|  | CH12 | -38 | -24 | 71 | 4.91 | <0.001 | 3.52 | 0.020 |
| Primary somatosensory cortex | CH2 | -48 | -36 | 64 | 5.26 | <0.001 | - | - |
|  | CH9 | -57 | -23 | 55 | 3.38 | 0.012 | - | - |
|  | CH11 | -25 | -39 | 75 | 2.65 | 0.049 | - | - |
|  | CH10 | -61 | -11 | 45 | 2.79 | 0.038 | - | - |
| Contralesional |  |  |  |  |  |  |  |  |
| Pre-motor and supplementary motor cortex | CH34 | 35 | 0 | 66 | 3.28 | 0.015 | 4.28 | 0.005 |
|  | CH33 | 47 | -12 | 63 | 3.89 | 0.006 | - | - |
|  | CH18 | 25 | -13 | 75 | 2.81 | 0.038 | - | - |
| Primary motor cortex | CH17 | 38 | -24 | 72 | 3.73 | 0.007 | 4.34 | 0.005 |
|  | CH16 | 14 | -26 | 80 | - | - | 3.32 | 0.021 |
| Somatosensory association cortex | CH6 | 37 | -51 | 69 | - | - | 2.91 | 0.046 |

MNI = Montreal Neurological Institute

The one-sample t-test was performed to compare β values with 0, and the false discovery rate (FDR) method was applied for multiple comparison correction to determine the activation status of each channel at the group level.

*S2.3. Spatial distribution of peak activation channels*

The location of the peak activation channel within the contralesional hemisphere for each participant was identified. The frequency with which specific channels served as the peak activation site was then tallied for responders and non-responders separately. Table S3 details the specific fNIRS channels that were identified as the peak activation site for individual patients, categorized by response group. It presents the channel number, its MNI coordinates, the associated ROI, and the count (frequency) of patients in each response group for whom that channel was the peak.

**Table S3.** Peak activation channels and frequencies in responders and non-responders

| Brain region | Channel | MNI coordinates, mm | | | Responders  (n=32) | Non-responders  (n=28) |
| --- | --- | --- | --- | --- | --- | --- |
|  |  | x | y | z |  |  |
| Pre-motor and supplementary motor cortex | CH18 | 25 | -13 | 75 | 4 (12%) | 4 (12%) |
|  | CH34 | 35 | 0 | 66 | 6 (19%) | 2 (7%) |
|  | CH35 | 54 | 1 | 53 | 1 (3%) | 4 (14%) |
|  | CH38 | 65 | 2 | 34 | 0 (0%) | 1 (4%) |
|  | CH46 | 23 | 12 | 70 | 1 (3%) | 1 (4%) |
|  | CH31 | 13 | 0 | 75 | 1 (3%) | 1 (4%) |
| Primary motor cortex | CH16 | 14 | -26 | 80 | 3 (9%) | 7 (25%) |
|  | CH33 | 47 | -12 | 63 | 6 (19%) | 0 (0%) |
|  | CH17 | 38 | -24 | 72 | 4 (12%) | 1 (4%) |
| Primary somatosensory cortex | CH7 | 48 | -35 | 63 | 2 (6%) | 0 (0%) |
|  | CH15 | 26 | -38 | 76 | 1 (3%) | 3 (11%) |
|  | CH21 | 63 | -10 | 45 | 1 (3%) | 0 (0%) |
|  | CH19 | 58 | -23 | 55 | 1 (3%) | 0 (0%) |
| Somatosensory association cortex | CH4 | 14 | -52 | 77 | 1 (3%) | 3 (11%) |
|  | CH6 | 37 | -51 | 69 | 0 (0%) | 1 (4%) |

MNI = Montreal Neurological Institute

*S2.4 Consistency of activation patterns across task parameters*

A post-hoc analysis was conducted to ensure that the spatial signature of cortical activation was not dependent on the specific pacing of the wrist movement task (slow: 0.25 Hz vs. fast: 1 Hz). Group-level activation maps were generated for each condition separately (Figure S1).

**Fig. S1.** Pretreatment cortical activation maps for different wrist movement conditions and response subgroups. Group-level cortical activation maps (t-statistics) during affected wrist extension under slow (0.25 Hz, Condition 1) and fast (1 Hz, Condition 2) pacing conditions are shown for all patients in the 1 Hz rTMS cohort (n=60), as well as for responders (n=32) and non-responders (n=28) separately. The contrast maps (Condition 2 > Condition 1) in the right column show no statistically significant differences in activation between the two movement conditions at the group level (FDR-corrected p > 0.05 for all comparisons). This indicates that the spatial characteristics of cortical activation were consistent across task parameters, justifying the combination of both conditions for the primary analysis.


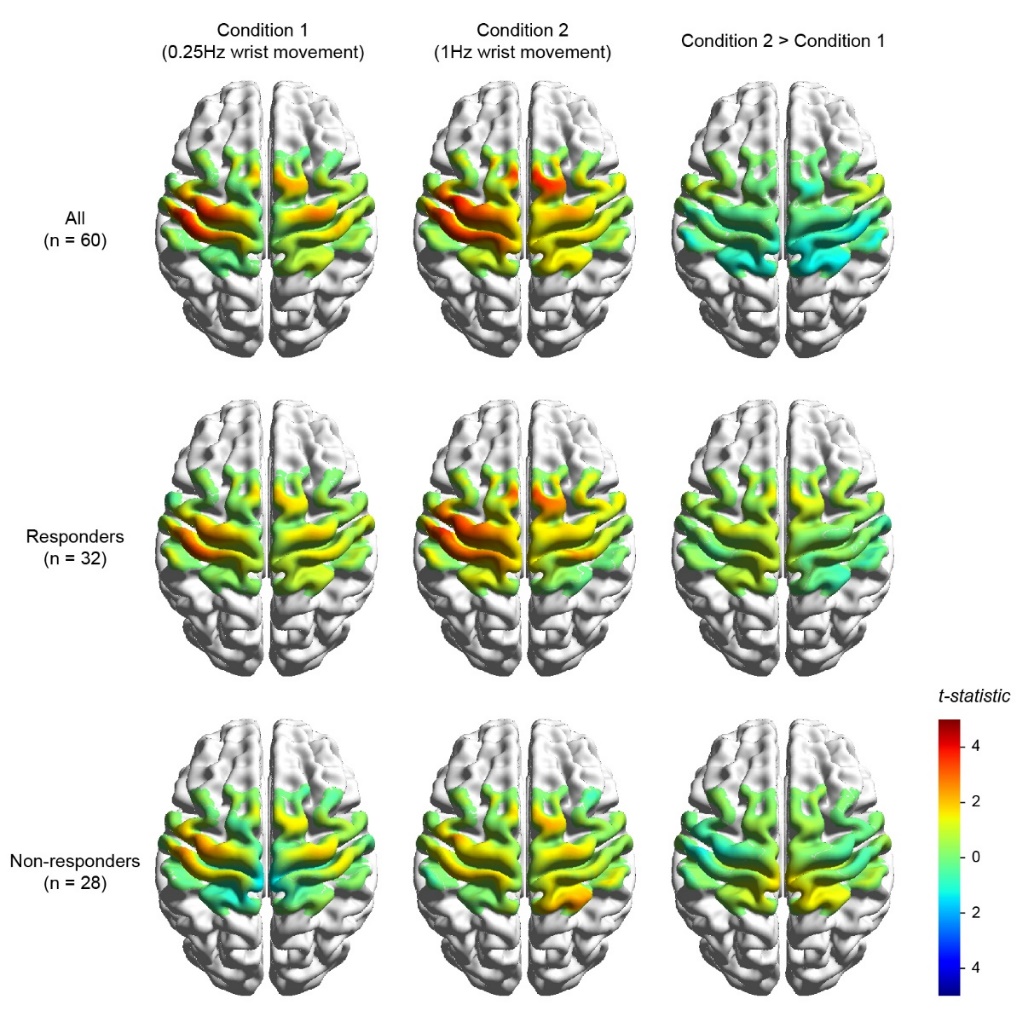


*S2.5 Robustness of the primary finding: Sensitivity analyses*

To assess the robustness of the primary finding—the significant association between activation distance and treatment response—sensitivity analyses were performed using alternative methods for handling missing data from the four participants who withdrew before the 4-week assessment. Table S4 summarizes the results of the sensitivity analyses, comparing the findings from the primary analysis (which used the LOCF method) with those from the MICE and the Complete-Case Analysis. The adjusted Odds Ratios (ORs) and their 95% Confidence Intervals (CIs) for the association between a 10-mm increase in activation distance and treatment response are presented for each method. The consistency of the results across all three approaches (primary OR=0.40, MICE OR=0.43, Complete-Case OR=0.44) strongly indicates that the identified association is robust and not sensitive to the method used to handle the limited missing data.

**Table S4.** Results of sensitivity analyses examining the association between activation distance and treatment response

| Analysis Method | Univariate | | | Multivariate | | |
| --- | --- | --- | --- | --- | --- | --- |
|  | unadjusted OR | 95%CI | p-value | adjusted OR | 95%CI | p-value |
| Primary Analysis (LOCF) | 0.32 | 0.16-0.64 | 0.001 | 0.40 | 0.17-0.83 | 0.013 |
| Sensitivity Analysis (MICE) | 0.33 | 0.16-0.68 | 0.003 | 0.43 | 0.20-0.90 | 0.027 |
| Sensitivity Analysis (Complete-Case) | 0.34 | 0.17-0.68 | 0.002 | 0.44 | 0.18-0.88 | 0.020 |

**References**

Buuren, S.V., Groothuis-Oudshoorn, K., 2011. **mice** : Multivariate Imputation by Chained Equations in *R*. J. Stat. Soft. 45. https://doi.org/10.18637/jss.v045.i03

Heinze, G., 2006. A comparative investigation of methods for logistic regression with separated or nearly separated data. Statistics in Medicine 25, 4216–4226. https://doi.org/10.1002/sim.2687

Little, R.J., D’Agostino, R., Cohen, M.L., Dickersin, K., Emerson, S.S., Farrar, J.T., Frangakis, C., Hogan, J.W., Molenberghs, G., Murphy, S.A., Neaton, J.D., Rotnitzky, A., Scharfstein, D., Shih, W.J., Siegel, J.P., Stern, H., 2012. The Prevention and Treatment of Missing Data in Clinical Trials. N Engl J Med 367, 1355–1360. https://doi.org/10.1056/NEJMsr1203730
